# Short-Term Medical Costs of a VHA Health Information Exchange: A CHEERS-Compliant Article

**DOI:** 10.1097/MD.0000000000002481

**Published:** 2016-01-15

**Authors:** Dustin D. French, Brian E. Dixon, Susan M. Perkins, Laura J. Myers, Michael Weiner, Allan J. Zillich, David A. Haggstrom

**Affiliations:** From the Department of Ophthalmology and the Center for Healthcare Studies, Feinberg School of Medicine, Northwestern University, and the Veterans Affairs Health Services Research and Development Service, in Chicago, IL (DDF); Department of BioHealth Informatics, Indiana University School of Informatics and Computing, VHA Health Services Research and Development Center for Health Information and Communication, and Regenstrief Institute, Inc., (BED); Department of Biostatistics, Indiana University School of Medicine, (SMP); Center for Health Information and Communication, Department of Veterans Affairs, Veterans Health Administration, Health Services Research and Development Service; Division of General Internal Medicine, Department of Medicine, Indiana University School of Medicine, and Regenstrief Institute, Inc. (LJM, MW, DAH); and Center for Health Information and Communication, Department of Veterans Affairs, Veterans Health Administration, Health Services Research and Development Service,; Regenstrief Institute, Inc.; Indiana University Center for Health Services and Outcomes Research, Indianapolis, IN; College of Pharmacy, Purdue University, West Lafayette, IN (AJZ).

## Abstract

The Virtual Lifetime Electronic Record (VLER) Health program provides the Veterans Health Administration (VHA) a framework whereby VHA providers can access the veterans’ electronic health record information to coordinate healthcare across multiple sites of care. As an early adopter of VLER, the Indianapolis VHA and Regenstrief Institute implemented a regional demonstration program involving bi-directional health information exchange (HIE) between VHA and non-VHA providers.

The aim of the study is to determine whether implementation of VLER HIE reduces 1 year VHA medical costs.

A cohort evaluation with a concurrent control group compared VHA healthcare costs using propensity score adjustment. A CHEERs compliant checklist was used to conduct the cost evaluation.

Patients were enrolled in the VLER program onsite at the Indianapolis VHA in outpatient clinics or through the release-of-information office.

VHA cost data (in 2014 dollars) were obtained for both enrolled and nonenrolled (control) patients for 1 year prior to, and 1 year after, the index date of patient enrollment.

There were 6104 patients enrolled in VLER and 45,700 patients in the control group. The annual adjusted total cost difference per patient was associated with a higher cost for VLER enrollees $1152 (95% CI: $807–1433) (*P* < 0.01) (in 2014 dollars) than VLER nonenrollees.

Short-term evaluation of this demonstration project did not show immediate reductions in healthcare cost as might be expected if HIE decreased redundant medical tests and treatments. Cost reductions from shared health information may be realized with longer time horizons.

## INTRODUCTION

Health Information Exchange (HIE) allows health care professionals and patients to access a patient's medical information electronically across healthcare sites and institutions. HIE has the potential to lower costs by improving medication reconciliation, reducing redundant or duplicative testing, and more efficiently coordinating care among multiple providers. For the Veterans Health Administration (VHA), the potential cost savings could be large because most VHA patients receive at least some care from outside providers and consequently receive testing and treatment that are not in the VHA system and thus not recorded in VHA electronic health records.^1–3^ The VLER (Virtual Lifetime Electronic Record) Health Program allows VHA and non-VHA health care providers to share health information from a veteran's health record electronically (bidirectionally).^4,5^ Prior studies in the US and abroad have suggested potential cost savings of HIE in emergency settings.^6–8^ Here we examine the association of VHA total cost savings of the health information exchange (HIE) for 1-year postimplementation at the Roudebush VHA medical center, Indianapolis, Indiana.

## METHODS

### Intervention

Through the VLER HIE program, the VHA electronically shared parts of veterans’ electronic health records with providers participating in the Indiana Health Information Exchange (IHIE). IHIE provides services based upon the Indiana Network for Patient Care (INPC) developed by the Regenstrief Institute.

The VLER HIE program builds upon the technical and policy standards of the eHealth Exchange, formerly the Nationwide Health Information Network.^4,5^ Implementation of the Indianapolis VLER demonstration program was overseen by a leadership group in VHA national offices and a local implementation team. Members of the local implementation team included the Chief Health Informatics Officer, Release of Information department, clinician champions, and a community coordinator who established veteran educational processes, as well as supported provider training and patient recruitment.

Patients who visited the Indianapolis VHA to attend clinic or go to the release of information office were approached by medical center staff, including nurses in VHA primary care clinics, and asked to enroll in VLER. Patients were given informational handouts developed by the VHA national office, and answered any questions. VLER was a voluntary opt-in program and there was no financial incentive offered to veterans for enrollment. Recruitment efforts did not involve any mailings.

Although recruitment was done in-person, both VLER participants and controls needed at least 2 outpatient visits or 1 inpatient episode to be included in our analytic cohort. This study was approved by both the Indiana University (Indianapolis, Indiana) and the Hines VHA (Chicago, Illinois) institutional review boards and the VHA research and development committee for ethics, human subject, and privacy protection assurance. All patients were consented and opted-in per our institutional review board approved protocol.

### Design and Data Sources

**FIGURE 1:**
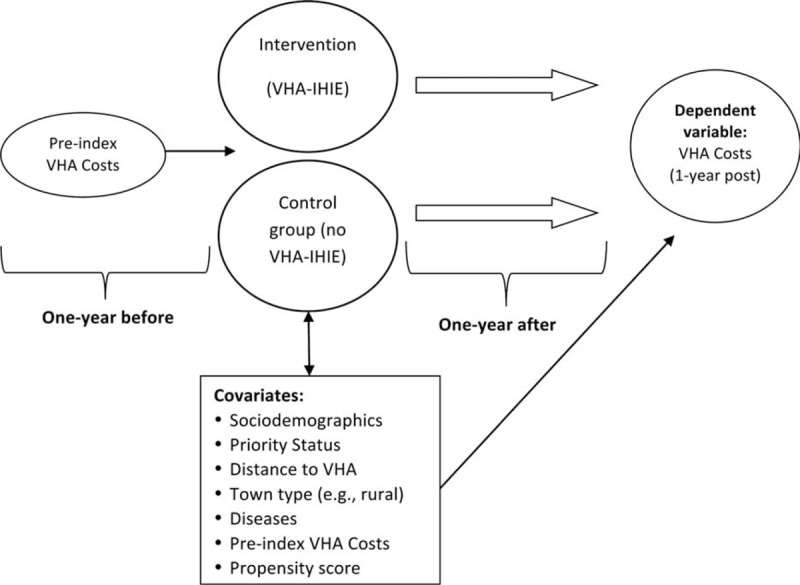
Study design.

The study design consists of a retrospective cohort with concurrent control group (Figure 1) with comparison of arms adjusted with propensity-score technique. The main outcome is 1 year total VHA medical costs (in 2014 dollars) from a VHA cost perspective obtained through the VHA's decision support system. Complete documentation of VHA cost data sets is available on-line (http://www.herc.research.va.gov/files/BOOK_621.pdf). Our cost datasets included aggregated inpatient and outpatient costs with components that may include emergency room, pharmacy, radiology, and so on. As this is a 1-year evaluation of medical costs and not an overall program evaluation, a social discount rate did not apply. Subjects were selected for inclusion by satisfying a minimum level of utilization of VHA services (2 outpatient visits or 1 inpatient episode prior to enrollment). Administrative data was extracted for the cohort of veterans for 1 year pre- and post enrollment date in VLER HIE from the VHA only. Specific data sets used included VHA's decision support system cost datasets, Medicare's Diagnostic Cost Groups, Hierarchical Condition Categories (DCG/HCCs), and VHA hospitalization and outpatient datasets.

For analysis, veterans were split into those who enrolled and a control group of those not enrolled. Those who did not enroll in the information exchange were given a default enrollment date. Covariate adjustment was based on factors that could impact enrollment in VLER and VHA post enrollment costs, including pre-index VHA medical costs, age at enrollment, gender, black race, marital status, distance to nearest VHA facility, urban versus rural characteristics, VHA priority status, Medicare's Diagnostic Cost Groups, and Hierarchical Condition Categories (DCG/HCCs) (Figure 1). Many of these variables are highly predictive of utilizing care in VHA as opposed to care in non-VHA setting.^1,2^

The DCG/HCC comorbidity adjustment was carried out with nationally maintained VHA data sets using the CMS methodology, from which we selected 88 of the 184 comorbidities relevant to an adult population.^9,10^ The hierarchical condition categories included HIV/AIDS, bacterial infections, cancer (all types), heart failure, ischemic heart disease, cardiac arrhythmias, osteoporosis, chronic renal disease, chronic liver disease, cerebrovascular disease, diabetes mellitus and various complications, pneumonias, asthma, chronic lung disease, depression, dementias, and central nervous system disorders.

Distance to nearest VHA facility was measured in miles as a Euclidean distance between the location of healthcare facility and the centroid of the zip code of the patient's residence and was obtained from the VHA Planning Systems Support Group.^2,8^ Independent of distance, the sociocultural characteristics of patients who reside in cities versus towns could also influence costs; therefore, Rural Urban Commuting Area (RUCA) codes, version 2.0 were used to identify type of town by the patient zip code.^11^ We used the University of Washington Categorization-A, which collapses the 33 RUCA codes into 4 groups (urban, large rural city/town, small rural town, isolated small rural town) and used the isolated small rural town as the reference group.

Veteran patient financial incentives may also impact the utilization of VHA services. Priority levels of 1 to 8 are assigned to all VHA enrollees based on service-connected disability and income level. The priority level influences the amount of copayment for VHA services. We created 4 groups for our analysis: priority 1 and 4 (catastrophically disabled), priority 2, 3, and 6 (moderate disability), priority 5 (Medicaid assistance/low income), and priority 7 and 8 (no service-connected disability), which served as the reference group.^2,12,13^

### Analysis

Multivariable regression models were constructed to examine our main outcome of total VHA healthcare costs in the 12-month study period. To address issues of skewness and violations of normality assumptions, a generalized linear model was tested with a negative binomial and gamma distribution with a log-link function.^14,15^ Potential imbalances between the VLER HIE and control groups and subsequent bias were addressed with propensity score adjustment.^16^

First, a logistic regression model with the key covariates described above was used to estimate the propensity for VLER enrollment (the propensity score). The propensity score was added as a covariate in a multiple regression model. Estimated parameters were obtained using the maximum likelihood technique, and adjusted results are presented with 95 percent confidence interval estimates. All analyses were conducted with the Statistical Analysis Software (SAS), version 9.3.

## RESULTS

Summary data of the cohorts is reported in Table 1. Both VLER and non-VLER groups were similar in terms of age and gender. However, the VLER cohort tended to^1^ live closer to a VHA facility (45.4 miles versus 68.0 miles, *P* < 0.01), often residing in more urban settings (83.8% vs 75.7%, *P* < 0.01), and^2^ have a significantly greater amount of chronic disease as captured by the hierarchical condition categories. The average cost per patient (unadjusted) in the post enrollment phase was $3386 higher for VLER than the non-VLER group (*P* < 0.01). Total adjusted cost for the VLER group with propensity score adjustment remained $1152 higher than the non-VLER group (95% confidence interval: $807–1433) (*P* < 0.01). The higher cost was driven by an aggregated outpatient cost that may include emergency room, pharmacy, and laboratory, radiology, and clinic visit related costs. We tested inpatient aggregated cost alone and found VLER was statistically insignificant at reducing costs.

**TABLE 1:**
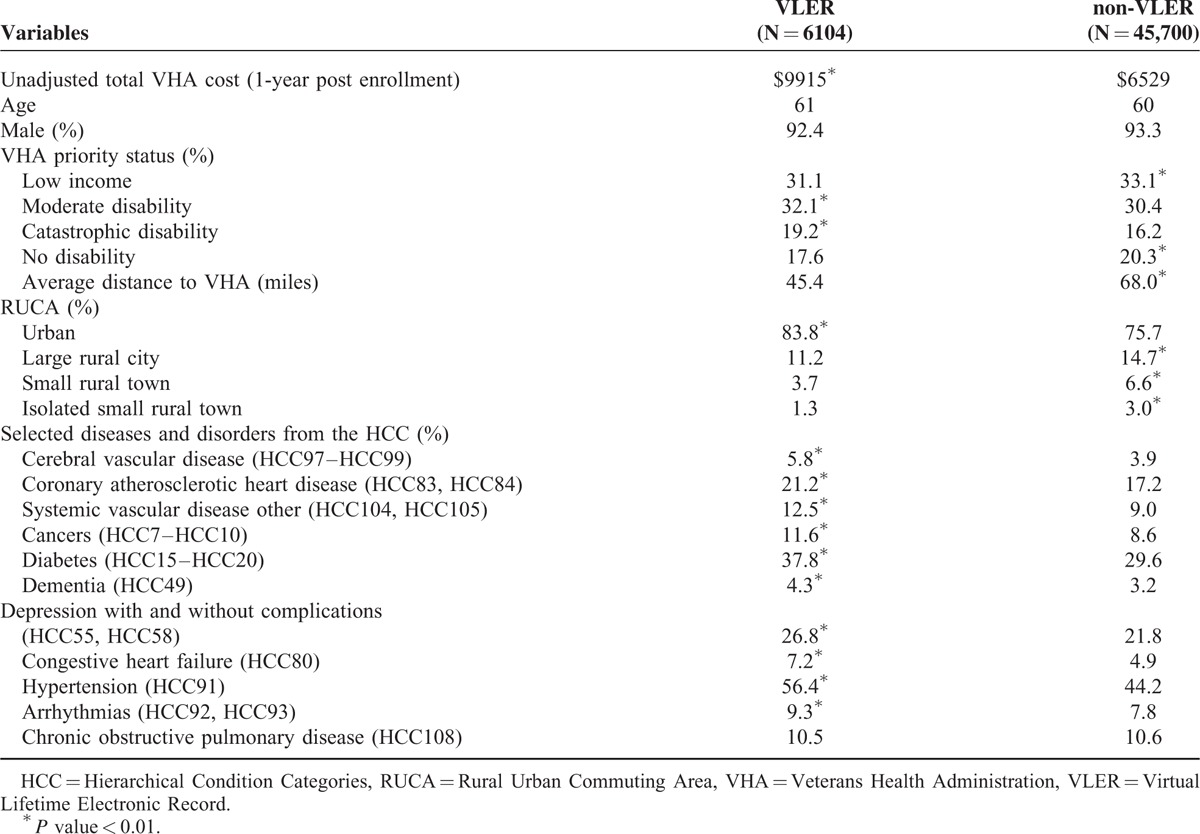
Cohort Summary Data

## DISCUSSION

This is the first study to report the associated impact of health information exchange (HIE) on VHA medical costs. The unique contribution of this study rests on the VHA system, which is not influenced by financial incentives that drive physician reimbursement like that in the private sector that could confound the study of HIE and its associated impact on costs. Within a 1-year time window, there was no reduction in medical costs from a VHA cost perspective, as might be expected if HIE reduced redundant testing at VHA facilities over the 1 year period of observation. A prior systematic review ^17^ found that, among HIE studies assessing costs, 56% (5/9) of observational studies and 100% (2/2) of experimental studies found a reduction in costs. Why then did our study of VHA HIE find increased costs among HIE enrollees?

First, although VLER HIE did not reduce VHA costs, it may have reduced healthcare costs overall by reducing costs among community exchange partners. We could not measure costs among community exchange partners and measured only a VHA cost accounting stance. Second, selective enrollment may have weakened internal validity as patients enrolled in VLER had greater illness severity and medical needs. Although we adjusted for comorbidity using the robust DCC/HCG methodology, residual confounding may have persisted.^18,19^ The risk for selection bias may have been increased due to patients being enrolled into the VLER on-site, thus, increasing the likelihood that veterans with greater care needs were recruited into the program. This is not just a potential limitation of this evaluation but *all* HIE evaluations of HIE programs requiring an opt-in process. Other methodological approaches were also tested including difference in difference specification, and various statistical models. These analyses failed to over-turn our current finding. Third, the higher costs of those with HIE may seem at first counterintuitive, but a very plausible explanation is simply that external medical information may have prompted VHA doctors to order additional tests and procedures that increase cost. To verify this explanation we are currently investigating order sets and quality measures with subgroups of diabetic patients with HIE, and the association with overall drug utilization, and imaging studies. These additional studies to be published at a later date are also producing a negative finding of HIE and suggest no reduction in medical utilization as a result of HIE. Given the mission of the VHA, it is entirely likely that any additional utilization and associated costs was deemed in the best interest of the veteran by his or her provider.

Finally, HIE technology implementation among providers may fall on the early phase of the adoption curve,^20^ and HIE cost reductions may be realized over a longer time horizon. Future research not only needs to address the time horizon needed to realize a reduction in medical cost but also assess the time needed for organizations to have a net economic savings (if any), accounting for the technology investment and support costs. Such delayed effects have been observed in changes to other healthcare systems, including quality improvement collaboratives.^21,22^ To address these limitations, future studies should consider randomized evaluations of cost, as well as weigh the changes in VHA costs against the quality of care delivered to veterans. Nonetheless, short-term evaluation of this demonstration project did not show reductions in healthcare costs as might be expected if HIE solely decreased redundant or duplicative testing and treatments in the VHA.
